# Prenatal serotonin reuptake inhibitor exposure and maternal depression symptoms are associated with altered fetal brain and placental development

**DOI:** 10.1038/s41386-026-02436-9

**Published:** 2026-05-09

**Authors:** Yao Wu, Nickie Andescavage, Katherine L. Wisner, Kushal Kapse, Jonathan Murnick, Julius S. Ngwa, Catherine Limperopoulos

**Affiliations:** 1 Developing Brain Institute, Children’s National Hospital, Washington, DC USA; 2 Division of Neonatology, Children’s National Hospital, Washington, DC USA; 3 Department of Diagnostic Imaging and Radiology, Children’s National Hospital, Washington, DC USA

**Keywords:** Risk factors, Outcomes research

## Abstract

Maternal mental health is associated with fetal neurodevelopment. Identifying effective treatments for maternal psychiatric conditions is a public health priority. SRIs (SSRIs and SNRIs) are commonly prescribed for prenatal mental health conditions; however, their impact on fetal brain development remains understudied. In this observational cohort study, we compared fetal brain and placental structures between SRI-exposed and unexposed pregnancies divided by categories of maternal depressive symptom severity from the Edinburgh Postnatal Depression Scale (EPDS). Pregnant women treated with SRIs and controls without mental illness or antidepressant exposure underwent fetal MRI studies between 20-40 weeks’ gestation. Fetal brain motion correction and 3D reconstructions were performed using slice-to-volume registration. Fetal brain volumes (cortical gray matter, white matter, deep gray matter, cerebellum, brainstem, and hippocampi) were quantified using deep learning-based segmentation with manual correction. Cerebral cortical folding measures included local gyrification index, sulcal depth, curvedness, and surface area. Placental volume and microstructure were assessed with T2-weighted and diffusion-weighted MRI, respectively. EPDS scores were categorized as low ( ≤ 4), moderate (5–9), and high ( ≥ 10). A total of 182 pregnant women were included [62 SRI-exposed (59 SSRIs, 3 SNRIs); 120 controls]. Notably, 29% of SRI-exposed women continued to report elevated depression. SRI-exposed fetuses had smaller hippocampal volumes and reduced cortical gyrification, curvedness, and surface area. Subgroup analysis of stratification by EPDS scores revealed that SRI-exposed fetuses had reduced hippocampal volumes compared to unexposed fetuses with low and moderate, but not high, EPDS scores, and reduced cortical curvedness compared to unexposed subgroups. Among unexposed subgroups, fetuses exposed to high maternal EPDS scores had smaller hippocampal volumes compared to those with low scores. Placenta volume and microstructural diffusion were increased in the SRI-exposed compared to the unexposed group. Larger placental volume was associated with larger total fetal brain volume, and higher placental diffusion was associated with larger fetal white matter and cerebellar volumes in the SRI-exposed group. These findings suggest that prenatal SRI exposure may be associated with altered fetal hippocampal volumes, cerebral cortical maturation, and placental volume and microstructural diffusion. The clinical significance and long-term neurodevelopmental consequences of these structural alterations remain unknown and are currently under study.

## Introduction

Maternal depression and anxiety are prevalent and impact up to 15% of women in the peripartum period [1–3]. Increasing evidence highlighting potential risks of untreated maternal depression includes complications such as miscarriage [4], preeclampsia [5], preterm delivery [6], and low birth weight [7]. Untreated depression during pregnancy is increasingly recognized as a risk factor adversely affecting offspring brain development, such as reduced cerebral and cerebellar gray matter volumes, increased cerebral cortical gyrification, and altered amygdala and hippocampal volumes as well as altered brain microstructure and functional connectivity [8–14]. Additionally, adverse child neurodevelopmental outcomes such as cognitive, language, learning, memory, social-emotional problems, and neuropsychiatric dysfunction [15–17] are reported after prenatal exposure to maternal mental health conditions, underscoring the importance of effective treatment strategies for both mother and child.

Selective serotonin reuptake inhibitors (SSRIs) and serotonin-norepinephrine reuptake inhibitors (SNRIs) are among the most commonly prescribed medications for managing mental illness during pregnancy [18]. Although both classes are generally considered low-risk choices for use during pregnancy [19, 20], they have potential risks. Women treated with SSRIs during pregnancy are at elevated risk for preterm birth and growth restriction [21, 22]. Brain MRI studies suggest that prenatal SSRI exposure is associated with alterations in brain structure and connectivity in the offspring, including increased gray matter volume in the right amygdala and right insula, as well as increased structural connectivity between the right amygdala and right insula in infants [23]. Higher connectivity in putative auditory resting-state networks [24] and lower fractional anisotropy, increased mean and radial diffusivity for multiple white matter fiber bundles have been reported in newborns [25]. Prenatal SSRI exposure has also been associated with reduced cerebral gray matter volume and steeper increases in amygdala and fusiform gyrus volumes in children aged 7–15 years [26]. Additionally, prenatal SSRI exposure may be associated with altered neurodevelopment and behavior in offspring, such as attenuated pain response in infants, and increased likelihood of anxiety or depression in adolescence [27–32]. Moreover, while placental dysfunction is implicated in preterm birth and growth restriction [33, 34], its relationship to maternal depression and SSRI exposure remains poorly understood. Understanding the impact of prenatal SSRI exposure on fetal brain and placental development is imperative.

We investigated the effects of prenatal exposure to SSRIs and SNRIs, collectively referred to as SRIs, on fetal brain volumes, cortical folding, and placental development. Given that the effects of SRI exposure are likely to be confounded by the severity of maternal psychiatric illness [35], maternal Edinburgh Postnatal Depression Scale (EPDS) scores were included as a continuous covariate in the main analyses to account for variance attributable to depressive symptoms. Additionally, we conducted subgroup analyses using a categorical approach (low, moderate, high EPDS) to examine whether fetal brain and placental differences between SRI-exposed and unexposed pregnancies varied across levels of maternal depressive symptoms which allowed assessment of whether observed differences were related to SRI exposure, maternal depressive symptoms, or both.

## Participants and methods

### Study design

This study included pregnant individuals undergoing treatment with SRIs, confirmed via medical records. A comparison group consisted of pregnant women without a current or lifetime episode of mental illness and without antidepressant treatment. Women were eligible if record reviews confirmed normal fetal ultrasounds and biometry studies. We excluded: (1) fetuses with known or suspected congenital infection, dysmorphic features, or dysgenetic lesions, or documented genetic or chromosomal abnormalities; and (2) pregnant women with multiple pregnancies or contraindications to MRI. Fetal brain and placenta MRI studies were performed between 20 and 40 weeks of gestation. This study was approved by the institutional review board at Children’s National Hospital and written informed consent was obtained by all participants prior to enrollment.

### Prenatal maternal depression

The Edinburgh Postnatal Depression Scale (EPDS) [36] was used to quantify depressive symptoms and was completed on the day of the MRI visit. The EPDS is the most commonly used screening tool during the perinatal period [37]. It is a 10-item questionnaire (range: 0–30) designed to measure the severity of symptoms over the prior 7 days. An EPDS score ≥10 is a positive screen for depression during pregnancy [38, 39]. We assigned EPDS scores into categories of ≤4, 5–9, and ≥10 as low, moderate, and high symptom levels, respectively. These clinically relevant cutoffs are well established and commonly used in obstetric and psychiatric studies [38–40].

### Fetal brain MRI acquisition

Fetal brain T2-weighted MRI was performed using a 1.5 T GE DISCOVERY MR450 scanner with an 8-channel receiver coil or a 3.0 T Siemens MAGNETOM Vida scanner with up to 48-channel surface receive coil system. Scanning protocols were harmonized between 1.5 T and 3.0 T scanners. At 1.5 T, the protocol included multiplanar single shot fast spin echo (SSFSE) acquisitions [echo time (TE): 160 ms; repetition time (TR): 1100 ms; flip angle (FA): 90°; field of view (FOV): 32×32 cm]. At 3.0 T, multi-plane multi-phase Half-Fourier acquisition single-shot turbo spin-echo (HASTE) T2-weighted images were performed (TE: 107 ms; TR: 1500 ms, FOV: 33 × 33 cm). For both 1.5 T and 3.0 T, imaging parameters included a matrix of 256 × 192, 2-mm slice thickness, and 0-mm slice gap. Interleaved acquisition, including odd and even slices, was performed to avoid slice crosstalk. For each subject, three T2w images were collected from each of axial, sagittal, and coronal planes. Participants were scanned during free breathing without the use of sedation or contrast agents.

### Fetal brain reconstruction

After acquisition, motion-corrupted stacks of 2-dimensional slices from coronal, sagittal, and axial planes were reconstructed into a 3-dimensional image (Supplementary Fig. 1). In this step, the fetal brain in MRI slices were automatically detected using You Only Look Once (YOLO) [41], a deep convolutional neural network designed for object detection. After brain detection, a parallel slice-to-volume reconstruction method with evaluated point-spread functions was applied to remove motion and reconstructed brain slices into a 3D image [42]. The reconstructed brain was rigidly registered to an in-house developed fetal brain atlas from 18 to 37 gestational weeks for reorientation using FSL FLIRT [43]. After brain reorientation, the image with 0.86 × 0.86 × 0.86 mm^3^ resolution was used in the following measures.

### MRI volumetric analysis

**Fig. 1:**
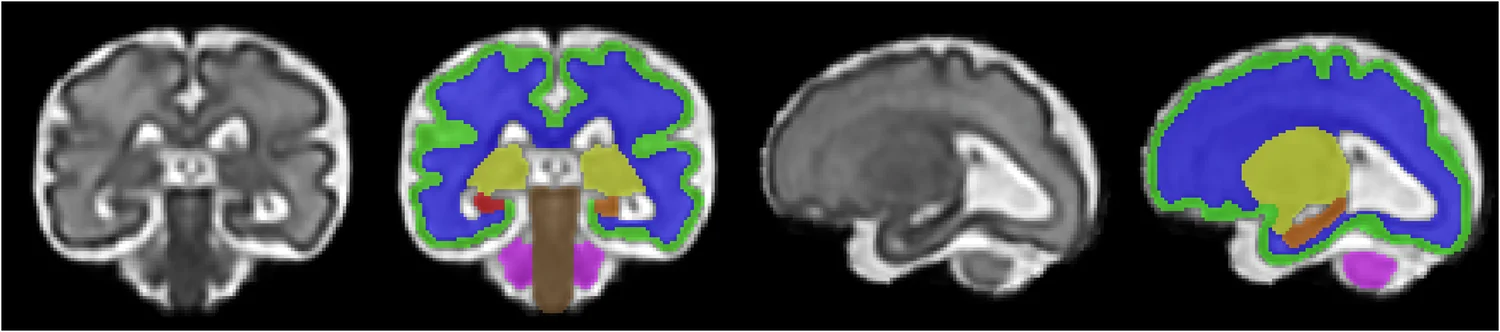
Fetal brain segmentation. Segmentation of cortical gray matter (green), white matter (blue), deep gray matter (yellow), cerebellum (violet), brainstem (brown), left hippocampus (orange), and right hippocampus (red) of a reconstructed fetal brain image at 29.7 gestational weeks.

Volumes of cortical gray matter, white matter, deep gray matter, cerebellum, brainstem, and left and right hippocampi were automatically segmented using a 3D U-Net based model (Fig. 1) [44]. 3D U-Net has demonstrated strong performance in medical image segmentation and has been validated for use in fetal brain image segmentation [44, 45]. Automatic segmentations were manually corrected using ITK-SNAP. A neuroradiologist with over 20 years’ experience in reading fetal MRI studies assisted with anatomical localization of these brain structures on MRI images. All structures were manually corrected by the same experienced rater, and 20% scans were randomly chosen and corrected by a second experienced rater. Inter-rater reliabilities using intraclass correlation coefficient for all measured regions were higher than 0.95. Raters were blinded to cohort status.

### Cerebral cortical folding

The inner surface of cortical gray matter was used to measure the cortical folding [46]. We analyzed: (1) Local gyrification index, the ratio between the cortical surface area and the corresponding area on cerebral hull surface within a sphere centered at each surface vertex [47]. (2) Sulcal depth, the distance from each vertex on the cortical surface to the nearest point on the cerebral hull surface [48]. (3) Curvedness, a measurement of the intensity of surface curvature [49]. (4) Surface area, the total cortical surface area calculated by summing the areas of all triangular surface meshes [47].

### Placenta MRI acquisition

Placenta imaging was acquired on the same scanner along with fetal brain MRI. At 1.5 T, T2-weighted sequence was acquired using multiplanar SSFSE (TR: 1100 ms; TE: 160 ms). At 3.0 T, HASTE T2-weighted images were acquired (TR: 1500 ms; TE: 107 ms). For both 1.5 T and 3.0 T, imaging parameters included a field of view 42 × 42 cm, 4-mm slice thickness, and 0-mm slice gap. Diffusion-weighted imaging (DWI) was performed using pulsed gradient spin echo (TR: 8000 ms, TE: 53.8 ms, matrix size 96×96, FOV: 42 × 42 cm, 4-mm slice thickness) with b-values: 0, 25, 50, 114, 243, 500, 543, 800, 900 sec/mm^2^, diffusion time of 25 ms. Scanning protocols were harmonized between 1.5 T and 3.0 T scanners.

### Placenta MRI processing

**Fig. 2:**
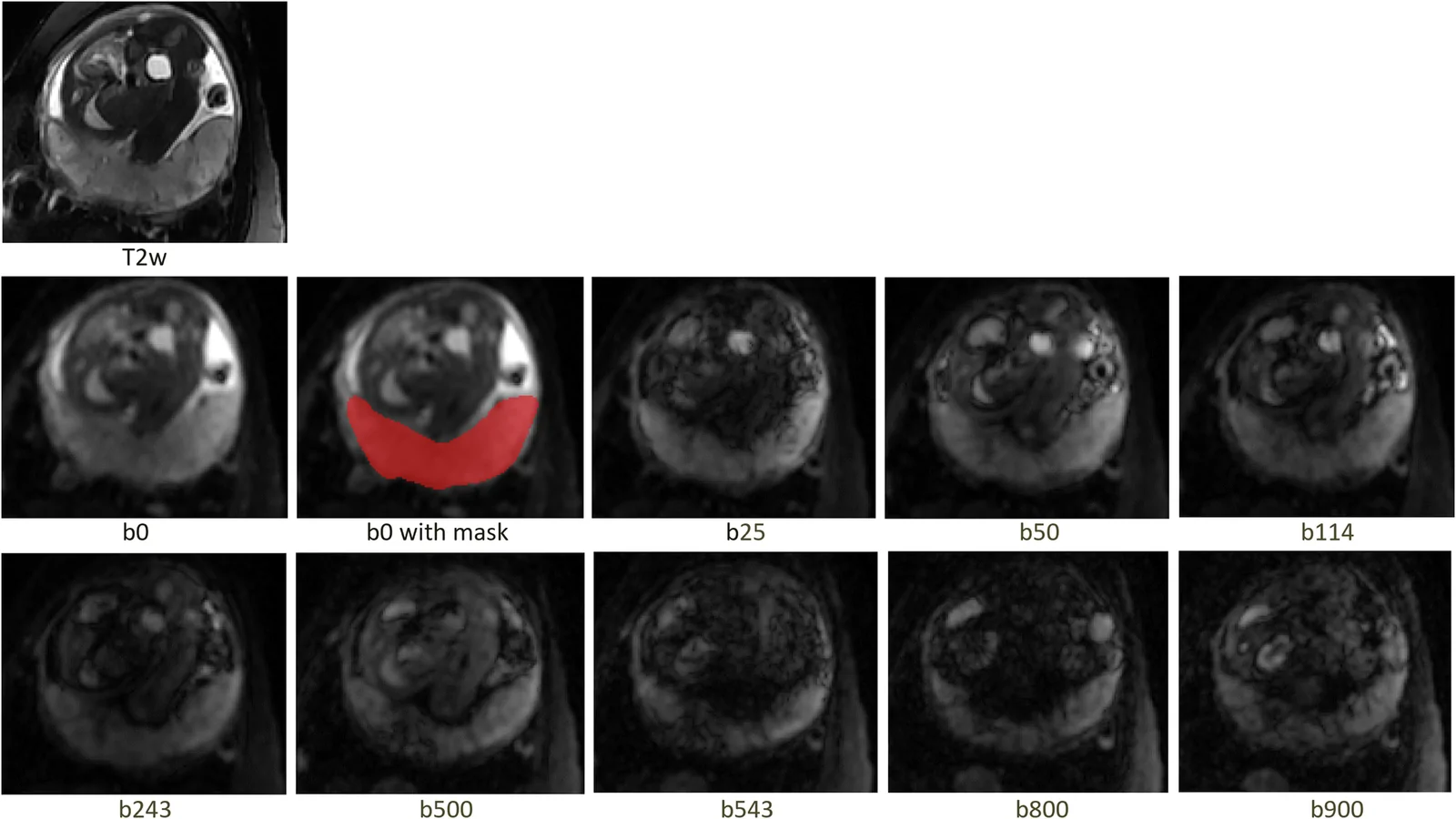
Placenta imaging. Placenta T2-weighted MRI scan (1st row) and diffusion weighted imaging scans with different b values (2nd and 3rd rows) of a subject at 30.29 gestational weeks.

The placenta was manually segmented on T2-weighted MR image for volumetric measurement. For microstructure diffusion, the placenta was manually segmented on the diffusion weighted image with b value of 0 s/mm^2^ using ITK-SNAP (Fig. 2). The corresponding T2-weighted image was used as anatomical reference during the manual segmentation. The placental mask was propagated to each b-value image and non-rigid registration was applied between b0 and subsequent b-value images for motion correction [50]. After that, the apparent diffusion coefficient (ADC) map was calculated by fitting of voxel-by-voxel using: S(b) = S_0_^(-b×ADC)^, where S(b) is the signal intensity at a certain b-value, S_0_ is the signal intensity at b = 0 [51]. ADC provides information about the magnitude of water molecule diffusion in tissues.

### Statistical analysis

Analysis was performed using SAS 9.3 and MATLAB R2024a. Subject characteristics in the SRI-exposed and unexposed groups were compared using *t* test for continuous variables and Fisher’s exact test for categorical variables. General linear model (GLM) was used to measure changes of fetal brain volumes, cortical folding, placenta measures, and maternal depression scales by gestational age (GA) and sex. Fetal brain measures between SRI-exposed group and unexposed controls were compared using analysis of covariance (ANCOVA), adjusting for GA at MRI, fetal sex, maternal depression score at MRI visit, and maternal weight at MRI. Fetal brain measures were further analyzed using ANCOVA, stratified by varying levels of maternal depression (EPDS ≤ 4, 5–9, ≥10) and adjusted for GA at MRI, fetal sex, and maternal weight at MRI. Placenta measures between SRI-exposed group and unexposed controls were compared using ANCOVA, adjusting for GA at MRI, maternal depression score at MRI visit, and maternal weight at MRI. Placental measures in the three categories of maternal depressive symptoms (EPDS ≤ 4, 5–9, ≥10) were analyzed using ANCOVA, adjusting for GA at MRI and maternal weight at MRI. *p* values in each table were adjusted for multiple testing based on the false discovery rate according to the Benjamini-Hochberg method [52], and adjusted *p* values ≤ 0.05 were considered significant.

## Results

### Demographics

Our cohort consisted of 182 participants: 62 with SRI (59 SSRIs, 3 SNRIs) exposure and 120 unexposed controls (Table 1). None of our enrolled participants reported receiving or undergoing psychotherapy. The mean (SD) [range] GA at MRI was 31.72 (4.05) [20.0-38.57] weeks for the SRI-exposed group and 32.04 (4.32) [23.57-39.71] weeks for controls, respectively. There were 27 (44%) and 67 (56%) of women carrying male fetuses for the SRI-exposed group and controls, respectively. Maternal weight at MRI was higher in the SRI group versus controls (86.21 vs 76.58 kg, *p* = 0.0001). Maternal alcohol use during pregnancy was higher in the SRI versus the unexposed group (16% vs 5%, *p* = 0.02). In both groups, more than half of women were non-Hispanic White. More than 90% and 84% of women were college graduates, and 82% and 84% reported professional employment in the SRI and control groups, respectively.

**Table 1:** Demographic and clinical characteristics.

Clinical Variables	SRI-exposed (*n* = 62)	Unexposed (*n* = 120)	*P* values
GA at MRI, mean (SD) [range], week	31.72 (4.05) [20.0–38.57]	32.04 (4.32) [23.57–39.71]	0.62
Male, N (%)	27 (44)	67 (56)	0.21
Maternal age, mean (SD) [range], year	35.81 (4.62) [22.0–44.49]	34.71 (5.87) [20.36–50.99]	0.17
Maternal weight at MRI, mean (SD) [range], kg	86.21 (16.25) [62.9–145.0]	76.58 (12.39) [53.60–119.0]	0.0001
Maternal smoking during pregnancy, N (%)	2 (3)	1 (1)	0.27
Maternal alcohol use during pregnancy, N (%)	10 (16)	6 (5)	0.02
Primigravida, N (%)	25 (40)	44 (37)	0.87
Primipara, N (%)	37 (60)	60 (50)	0.35
Maternal education, N (%)			0.12
≤High school	1 (2)	5 (4)	
Some college	2 (3)	11 (9)	
College graduate	13 (21)	37 (31)	
Graduate degree	43 (69)	64 (53)	
Unknown	3 (5)	3 (3)	
Maternal employment, N (%)			0.76
Professional	51 (82)	101 (84)	
Skilled/clerical/sales	1 (2)	4 (3)	
Semiskilled operator	0 (0)	3 (3)	
Unemployed/Homemaker	5 (8)	8 (7)	
Unknown	5 (8)	4 (3)	
Maternal race, N (%)			0.78
Asian/Pacific Islander	4 (6)	8 (7)	
Non-Hispanic Black	6 (10)	18 (15)	
Hispanic	5 (8)	12 (10)	
Non-Hispanic White	44 (71)	72 (60)	
Other or unknown	3 (5)	10 (8)	

*P* value based on *t* test for continuous variables and Fisher’s exact test for categorical variables.

### Maternal depressive symptoms

Maternal depression scores were significantly higher in the SRI-exposed group compared to unexposed controls (mean: 6.55 vs 4.75, *p* = 0.01). The proportion of EPDS score ≥10 was significantly higher in women treated with SRIs (29%) versus unexposed controls (13%). EPDS scores did not significantly change with increasing GA in controls (β = 0.03, *p* = 0.70). However, maternal scores decreased with advancing GA in the SRI-exposed group (β = –0.34, *p* = 0.02).

### Fetal brain volume and cortical folding

All measured fetal brain volume and cortical folding measures significantly increased with advancing GA in both the SRI and control groups (all *p* < 0.0001). In unexposed controls, males had significantly larger volumes in white matter (104.13 vs 99.94 cm^3^, *p* = 0.04), deep gray matter (16.03 vs 15.41 cm^3^, *p* = 0.03), and total brain (203.98 vs 197.71 cm^3^, *p* = 0.04), compared to females. In the SRI-exposed group, males had larger total brain volume compared to female fetuses (203.40 vs 187.59 cm^3^, *p* = 0.01). Left hippocampal volumes were smaller than the right hippocampus in both SRI-exposed (0.46 vs 0.50 cm^3^, *p* = 0.002) and control (0.52 vs 0.56 cm^3^, *p* < 0.0001) groups. Fetal cerebral cortical folding measures did not significantly differ in males and females.

### Placenta volume and microstructure measures

The placental volume significantly increased as GA increased in both groups (SRI: β = 39.91 cm^3^/week, *p* < 0.0001, controls: β = 30.07 cm^3^/week, *p* < 0.0001). In controls, placental ADC_D decreased with advancing GA (β = –0.00004, *p* = 0.03); however, no significant association was observed between placental ADC_D and GA in the SRI-exposed group. Placental measures did not significantly differ in pregnancies with male versus female fetuses.

### Fetal brain measures in SRI-exposed group vs controls

SRI-exposed fetuses showed significantly smaller left (0.45 vs 0.53 cm^3^, adjusted *p* = 0.0004) and right (0.48 vs 0.56 cm^3^, adjusted *p* = 0.0004) hippocampal volumes compared with controls (Table 2). Additionally, the SRI-exposed group showed reduced cerebral cortical local gyrification index (1.24 vs 1.28, adjusted *p* = 0.03), curvedness (0.21 vs 0.24 mm^–1^, adjusted *p* = 0.0004), and surface area (143.35 vs 151.36 cm^2^, adjusted *p* = 0.02) compared with controls (Table 2).

**Table 2:** Fetal brain volume and cortical folding in SRI-exposed group vs unexposed controls.

	SRI-exposed (*n* = 62)	Unexposed (*n* = 120)	Difference (95% CI)	Adjusted P^a^
**Brain volume (cm**^**3**^**)**				
Cortical gray matter	63.52	65.09	–1.57 (–4.47 to 1.34)	0.38
White matter	97.82	100.75	–2.93 (–7.05 to 1.19)	0.24
Deep gray matter	15.14	15.50	–0.35 (–0.82 to 0.11)	0.24
Cerebellum	9.31	9.74	–0.42 (–0.90 to 0.05)	0.16
Brainstem	4.33	4.31	0.02 (–0.11 to 0.15)	0.79
Left hippocampus	0.45	0.53	-0.07 (–0.10 to –0.04)	0.0004
Right hippocampus	0.48	0.56	–0.08 (–0.11 to –0.05)	0.0004
Total brain volume	195.64	197.70	–2.07 (–8.48 to 4.34)	0.57
**Cerebral cortical folding**				
Local gyrification index	1.24	1.28	–0.05 (–0.08 to –0.01)	0.03
Sulcal depth (mm)	1.65	1.69	–0.05 (–0.17 to 0.07)	0.53
Curvedness (mm^-1^)	0.21	0.24	–0.02 (–0.04 to –0.01)	0.0004
Surface area (cm^2^)	143.35	151.36	–8.01 (–13.74 to –2.27)	0.02

Results of least squares means from analysis of covariance, controlling for gestational age at MRI scan, sex, maternal weight at MRI, and maternal depression score at MRI visit.

^a^*P* values were adjusted for multiple testing based on the false discovery rate according to the Benjamini-Hochberg method.

Because maternal alcohol use during pregnancy was higher in the SRI compared to the unexposed group (Table 1), sensitivity analyses were performed by excluding participants with alcohol use during pregnancy from the comparison of fetal brain and placental measures between the SRI-exposed and unexposed groups, adjusting for the same covariates as in the main analyses. In this sensitivity analysis (Table S1), in addition to the main findings (in Table 2), fetal cerebellar volume also differed between the SRI-exposed and unexposed groups (mean: 9.15 vs. 9.67 cm³; 95% CI: −1.03 to −0.004) before adjustment for multiple comparisons. However, this difference was no longer statistically significant after correction for multiple comparisons (adjusted *p* = 0.10), consistent with the main findings in Table 2.

### Fetal brain measures in SRI-exposed group and controls relative to maternal depressive symptoms

For measures with significant differences between the SRI-exposed and unexposed groups (Table 2), additional subgroup analyses were conducted by stratifying based on maternal depression severity (Table S2). Both left and right hippocampal volumes in unexposed low- and moderate-EPDS groups were significantly larger than those in all three EPDS subgroups of SRI-exposed participants. Of note, fetuses in the unexposed high-EPDS group had smaller left and right hippocampal volumes compared to the unexposed low-EPDS group. Additionally, SRI-exposed fetuses in both the low and moderate EPDS subgroups showed reduced cortical curvedness compared to unexposed subgroups (Table S2). Fetal hippocampal volumes and cortical folding measures did not differ significantly across the low, moderate, and high EPDS subgroups within the SRI-exposed group (Table S2).

In the sensitivity analyses of the subgroup comparison after excluding participants with alcohol use during pregnancy (Table S3), the difference in fetal cortical curvedness between SRI-exposed fetuses with low and moderate EPDS scores and the unexposed group with high EPDS (in Table S2) remained significant before, but not after, adjustment for multiple comparisons (Table S3), suggesting that the observed subgroup differences in fetal cortical curvedness between SRI-exposed fetuses with low and moderate EPDS and the unexposed group with high EPDS may be less significant by the exclusion of participants with alcohol use during pregnancy.

### Placenta measures in SRI-exposed group vs controls

Placenta volumes (753.11 cm^3^ vs 656.00 cm^3^, adjusted *p* = 0.004) and ADC_D (0.004 vs 0.003, adjusted *p* = 0.048) were higher in SRI-exposed group vs unexposed controls (Table 3). Additional subgroup analyses stratified by maternal depression severity are presented in Table S4; however, the differences between subgroups were no longer significant after adjusting for multiple testing.

**Table 3:** Placenta measures in SRI-exposed group vs unexposed controls.

	SRI-exposed	Unexposed	Difference (95% CI)	Adjusted P^a^
Volume	753.11	656.00	97.11 (35.83 to 158.39)	0.004
ADC_D	0.004	0.003	0.0004 (0.000003 to 0.0008)	0.048

Results of least squares means from analysis of covariance, controlling for gestational age at MRI scan, maternal weight at MRI, and maternal depression score at MRI visit.

^a^*P* values were adjusted for multiple testing based on the false discovery rate according to the Benjamini-Hochberg method.

The placenta results in the sensitivity analyses (Tables S5 and S6) by excluding participants with alcohol use during pregnancy were consistent with the main findings in Table 3 and S4.

### Associations between placenta measures and fetal brain measures in SRI-exposed group

Larger placenta volume was associated with larger volumes in fetal deep gray matter (β = 0.002, *p* = 0.02) and total brain (β = 0.05, *p* = 0.005) (Table S7) as well as larger cortical surface area (β = 0.03, *p* = 0.03) (Table S8). Increased placenta ADC_D was associated with larger volumes in fetal cortical gray matter (β = 2493.92, *p* = 0.04), white matter (β = 6153.24, *p* = 0.01), cerebellum (β = 525.99, *p* = 0.01), and total brain (β = 6886.81, *p* = 0.03) as well as increased cortical gyrification index (β = 18.53, *p* = 0.03) and surface area (β = 6595.05, *p* = 0.008) (Table S8). The associations between placental volume and fetal total brain volume, as well as between placental ADC_D and fetal white matter and cerebellar volumes, remained significant after adjusting for multiple testing (Table S7).

## Discussion

We report that altered fetal brain development may be associated with both SRI exposure and elevated depression symptoms. SRI-exposed fetuses had smaller left and right hippocampal volumes, decreased cortical gyrification index, curvedness, and surface area. Notably, hippocampal volumes were reduced in SRI-exposed fetuses compared to unexposed fetuses in the low and moderate EPDS groups, and cortical curvedness was decreased in SRI-exposed fetuses in low and moderate EPDS groups compared to unexposed controls. Among unexposed fetuses, reduced hippocampal volumes were observed in those with high versus low maternal EPDS scores. Additionally, SRI-exposed pregnancies had increased placental volume and microstructural diffusion compared with unexposed pregnancies. Finally, within the SRI-exposed group, larger placental volume was associated with larger fetal total brain volume, and higher placental diffusion was associated with larger fetal white matter and cerebellar volumes.

We observed an unsettling 29% of women in the SRI-treated group continued to report EPDS scores ≥10. This rate is comparable to 32% reported in a similar sample of SRI-treated pregnant women [40] and significantly higher than the 13% observed in the unexposed group, which highlights the frequency of persistent depressive symptoms during pregnancy. The remaining SRI-exposed participants had low or moderate EPDS scores, suggesting remission or partial response. These findings may suggest inadequate SRI dosing or reductions in plasma concentrations due to increased drug clearance in pregnancy [53–55]. Importantly, this variation in symptom severity among SRI-treated participants provided an opportunity to explore potential differences in fetal and placental development associated with symptom control in SRI-treated mothers.

Importantly, we observed decreased left and right hippocampal volumes, as well as reduced cortical gyrification index, curvedness, and surface area in fetuses exposed to SRIs compared to unexposed controls after adjusting for maternal depression scales. Stratified analyses by maternal depression severity also suggested reduced hippocampal volumes and cerebral cortical curvedness in SRI-exposed subgroups (low, moderate, and high depression) compared to unexposed fetuses, indicating that these brain alterations may not be solely attributable to maternal depression, but also associated with SRI exposure across varying levels of maternal depression symptoms. Additionally, we found no significant differences in fetal hippocampal volumes when comparing SRI-exposed subgroups to unexposed fetuses with high maternal depression. This finding raises the possibility that high maternal depressive symptoms alone may adversely affect fetal hippocampal development to a similar extent as SRI exposure. The observed differences in fetal brain development may also reflect underlying genetic or familial vulnerability factors rather than SRI exposure per se. Notably, among unexposed subgroups, fetuses of women with high symptoms had smaller left and right hippocampal volumes compared to those with low depression, consistent with prior findings [10, 12], suggesting that elevated depressive symptoms can impact fetal hippocampal development independent of SRI exposure. In contrast, hippocampal volumes did not significantly differ across SRI-exposed subgroups with low, moderate, and high EPDS scores, which suggest that prenatal SRI treatment may mitigate the associations between elevated maternal depression and reduced fetal hippocampal volumes observed in the unexposed group. Therefore, in the presence of high depressive symptoms, the observed brain structural differences likely reflect a combination of medication exposure and underlying high-level maternal depressive symptoms, making it challenging to identify the independent effect of SRI exposure alone. These findings underscore the difficulty of disambiguating the effects of SRI treatment from those of maternal psychiatric illness. While these structural differences may reflect alterations in fetal brain maturation, the clinical significance and long-term neurodevelopmental consequences remain uncertain. The hippocampus is critical for memory and emotional regulation, while cortical curvature, a marker of cortical folding, reflects brain maturation [56–58]. Longitudinal follow-up studies incorporating genetic and familial factors are needed to determine the relationship between early brain structural alterations and subsequent developmental outcomes.

The potential mechanisms underlying the effects of SRI exposure on fetal hippocampal and cortical folding development are complex and multifaceted. In addition to the confounding effects of maternal psychiatric illness, comorbid conditions, and shared genetic and environmental factors [35, 59, 60], SRIs cross the placenta and potentially influence fetal brain development by altering levels of critical neurotransmitters [61–63]. Indeed, changes in serotonin levels during critical periods of brain development could alter the formation of neural circuits and potentially lead to brain structural alterations and subsequent neuropsychiatric outcomes [64].

To better understand feto-placental-maternal relationships, we investigated in vivo placental development and observed that SRI-exposed pregnancies showed larger placental volume and higher microstructural diffusion than controls. The DWI ADC model quantifies tissue microstructure by measuring water diffusion, enabling detection of subtle changes undetectable by conventional imaging [65, 66]. Diffusion variations may reflect altered vascularization and intervillous space, indicating disrupted villous maturation and placental dysfunction [67, 68]. Such dysfunction has been implicated in adverse outcomes including preterm birth and growth restriction, conditions previously associated with prenatal SRI exposure [21, 22]. Prior MRI-based studies have demonstrated that larger placental volume is associated with increased fetal cerebral and cerebellar volumes in fetal growth restriction and healthy pregnancies [69], as well as with increased subcortical gray matter, total brain, and intracranial volumes in fetuses with congenital heart disease (CHD) [70]. In addition, placental hypoplasia, vascular malperfusion, and the presence of placental pathology have been associated with reduced fetal total intracranial volume, particularly in CHD [71]. To our knowledge, the current study is the first to show that prenatal SRI exposure is associated with increased placental volume and microstructural diffusion, and that these placental alterations are associated with fetal total brain, white matter, and cerebellar volumes in SRI-exposed pregnancies. Our findings may reflect a compensatory adaptation to enhance the transfer of nutrients and growth factors, potentially supporting fetal brain development. The observed associations between placental volume and diffusion with fetal brain volumes highlight the complex relationship between placental function and fetal brain development in the context of SRI exposure. This study focused on fetal life, a specific stage in development, and interpretations of functional effects of these alterations are premature. Reassuringly, a large, fully adjusted pharmacoepidemiologic study found that prenatally antidepressant-exposed and unexposed siblings had similar risks of neurodevelopmental disorders through age 14 years [72].

Limitations of this study deserve mention. First, we were unable to evaluate the impact of the amount, timing, and duration of SRI exposure on fetal brain and placenta measures, due to the lack of detailed data. Second, although GA at MRI was included as a covariate in all analyses, the span of imaging between 20 and 40 weeks’ gestation encompasses a wide range of dynamic neurodevelopmental changes. GA represents a relatively broad developmental marker and may not fully serve as a proxy for other age-related biological processes (e.g., cerebral blood flow or metabolic demand) not directly measured in the current study. Similarly, maternal depression itself, independent of SRI exposure, may be associated with reduced physical activity and altered fetal movement patterns, which were not assessed in the current study. Such unmeasured developmental, behavioral, or physiological factors may have influenced fetal brain and placental development. Third, none of the enrolled participants reported receiving or undergoing psychotherapy during pregnancy, which may have led to underrepresentation of individuals with milder depressive symptoms and who may have been managed with psychotherapy alone, indicating our SRI-exposed group may reflect women with more clinically severe or treatment-resistant symptoms, which may affect the generalizability of our findings and should be considered when interpreting the results. Fourth, our cohort predominantly consisted of high-income, well-educated women which limits the generalizability of our findings to pregnant women across diverse socio-demographic backgrounds. In addition, with only three SNRI-exposed participants, we were unable to compare SSRI- and SNRI-specific effects. Larger samples of SNRI-exposed pregnancies are needed to evaluate potential class-specific differences. Although we examined differences across maternal depression severity, subgroups with high depression scores had relatively small sample sizes, which may have limited the statistical power of these analyses. Larger samples will be needed to more definitively evaluate fetal brain and placental development in relation to treatment response (remission versus persistent symptoms) during prenatal SRI exposure. The significance of maternal depressive symptoms can also vary based on assigned cut-off scores; in this study, we selected cut-off scores which have been previously used for pregnant women [38–40]. Furthermore, one-timepoint EPDS score does not capture the cumulative burden or temporal variability of depressive symptoms across pregnancy. While the EPDS is a well-validated measure for perinatal depression, it does not incorporate other dimensions of maternal psychological distress, such as perceived stress or anxiety, which may independently or jointly influence fetal brain and placenta development. In this study, we focused on depressive symptoms, given that prenatal depression is the most common perinatal mental health condition [73, 74] and is typically treated with SRIs when pharmacotherapy is needed during pregnancy [75, 76]. A detailed lifetime and prenatal course of maternal mental illness would clarify the impact of maternal psychiatric symptoms, beyond depression alone, and SRI treatment on fetal brain and placental development. Due to the challenges in fetal MRI, 9% of scans were unusable following 3D reconstruction because of excessive fetal motion; however, this proportion is consistent with or superior to other fetal MRI studies [77]. While we adjusted for age at MRI, sex, maternal weight, and maternal depression scores in the model, our sample size limited our ability to adjust for additional potential confounding factors, such as maternal physical health and lifestyle factors. Lastly, alcohol use during pregnancy was assessed as a binary self-reported variable with limited information on timing or frequency, and its impact on the main findings needs further investigation in prospective studies. Future studies with larger cohorts (including both SSRI- and SNRI-exposed pregnancies), detailed longitudinal exposure data (including timing, duration, and dosage of medication use), and more specific physiological, behavioral, and maternal mental health measures (including stress, anxiety, and ability to function) across gestation are needed to further characterize the effects of prenatal SRI exposure and maternal psychological distress on fetal brain and placenta development. Longitudinal investigations are underway to explore the impact of fetal brain and placenta developmental changes associated with depression and SRI exposure on birth and long-term neurobehavioral outcomes.

## Conclusions

In summary, this study provides novel insights into the association between prenatal SRI exposure, depressive symptom severity, and fetal brain and placenta development. These findings underscore the use of novel research tools to understand the contributions of both exposure to depressive symptoms and SRI treatment during pregnancy, which advances clinical decision-making aimed at improving both maternal health and offspring neurodevelopmental outcomes. Considering the well-established risks of untreated maternal depression, decisions regarding prenatal antidepressant use should be guided by clinical indications and individualized risk-benefit assessments, rather than structural imaging findings alone. These results highlight the need for longitudinal studies to examine the potential effects of prenatal SRI exposure and maternal depression on offspring neurodevelopment.

## Supplementary information

Supplemental Material

## Data Availability

The datasets generated and analyzed during the current study are available from the corresponding author on reasonable request.
