# Supplementary material for: Prenatal serotonin reuptake inhibitor exposure and maternal depression symptoms are associated with altered fetal brain and placental development
Source: Neuropsychopharmacology. 2026 May 9;51(11):1999–2007. doi: 10.1038/s41386-026-02436-9 (PMC13598115; doi:10.1038/s41386-026-02436-9)
Supplement: Supplementary file 1 [file 41386_2026_2436_MOESM1_ESM.docx]

Supplemental Figure S1. Fetal brain reconstruction from 2D Single Shot Fast Spin Echo MRI slices of axial, coronal, and sagittal planes (left) to a single 3D image without motion (right).

Supplemental Table S1. Fetal brain volume and cortical folding in SRI-exposed group vs unexposed controls, excluding participants with prenatal alcohol exposure.

|  | **SRI-exposed (n=52)** | **Unexposed (n=114)** | **Difference (95% CI)** | **Adjusted P^a^** |
| --- | --- | --- | --- | --- |
| **Brain volume (cm^3^)** | | | | |
| Cortical gray matter | 62.65 | 64.89 | -2.24 (-5.31 to 0.83) | 0.20 |
| White matter | 97.32 | 100.69 | -3.36 (-7.88 to 1.15) | 0.20 |
| Deep gray matter | 15.04 | 15.44 | -0.408 (-0.91 to 0.09) | 0.19 |
| Cerebellum | 9.15 | 9.67 | -0.52 (-1.03 to -0.004) | 0.10 |
| Brainstem | 4.28 | 4.29 | -0.008 (-0.14 to 0.12) | 0.90 |
| Left hippocampus | 0.44 | 0.52 | -0.08 (-0.11 to -0.05) | 0.0004 |
| Right hippocampus | 0.47 | 0.55 | -0.08 (-0.11 to -0.05) | 0.0004 |
| Total brain volume | 194.58 | 197.02 | -2.45 (-9.39 to 4.50) | 0.53 |
| **Cerebral cortical folding** | | | | |
| Local gyrification index | 1.24 | 1.28 | -0.05 (-0.08 to -0.008) | 0.04 |
| Sulcal depth (mm) | 1.64 | 1.67 | -0.06 (-0.19 to 0.07) | 0.45 |
| Curvedness (mm^-1^) | 0.21 | 0.24 | -0.03 (-0.04 to -0.01) | 0.0004 |
| Surface area (cm^2^) | 143.49 | 151.76 | -8.27 (-14.41 to -2.13) | 0.03 |

Results of least squares means from analysis of covariance, controlling for gestational age at MRI scan, sex, maternal weight at MRI, and maternal depression score at MRI visit.

^a^ P values were adjusted for multiple testing based on the false discovery rate according to the Benjamini-Hochberg method.

Supplemental Table S2. Fetal brain measures in SRI-exposed group and unexposed controls with different prenatal maternal depression scales.

|  | **SRI-exposed** | | | **Unexposed** | | |
| --- | --- | --- | --- | --- | --- | --- |
| **EPDS** | **≤ 4**  (n=19) | **5-9**  (n=20) | **≥10**  (n=16) | **≤ 4**  (n=67) | **5-9**  (n=33) | **≥10**  (n=15) |
| **Brain volume (cm^3^)** | | | | | | |
| Left hippocampus | 0.43^a,b^ | 0.46^a^ | 0.44^a,b^ | 0.54^c^ | 0.52 | 0.48 |
| Right hippocampus | 0.45^a,b^ | 0.49^a,b^ | 0.47^a,b^ | 0.57^c^ | 0.55 | 0.51 |
| **Cerebral cortical folding** | | | | | | |
| Local gyrification index | 1.25 | 1.25 | 1.21 | 1.27 | 1.29 | 1.30 |
| Curvedness (mm^-1^) | 0.21^a,c^ | 0.21^a,b,c^ | 0.22 | 0.24 | 0.24 | 0.24 |
| Surface area (cm^2^) | 141.91 | 145.83 | 141.39 | 151.27 | 151.33 | 151.79 |

Results of least squares means from analysis of covariance, controlling for gestational age at scan, fetal sex, maternal weight at MRI.

^a^ Different from Unexposed fetuses with maternal EPDS ≤ 4 (adjusted p <0.05);

^b^ Different from Unexposed fetuses with maternal EPDS 5-9 (adjusted p <0.05);

^c^ Different from Unexposed fetuses with maternal EPDS ≥10 (adjusted p <0.05)

P values were adjusted for multiple testing based on the false discovery rate according to the Benjamini-Hochberg method.

Supplemental Table S3. Fetal brain measures in SRI-exposed group and unexposed controls with different prenatal maternal depression scales, excluding participants with prenatal alcohol exposure.

|  | **SRI-exposed** | | | **Unexposed** | | |
| --- | --- | --- | --- | --- | --- | --- |
| **EPDS** | **≤ 4**  (n=17) | **5-9**  (n=16) | **≥10**  (n=13) | **≤ 4**  (n=63) | **5-9**  (n=33) | **≥10**  (n=13) |
| **Brain volume (cm^3^)** | | | | | | |
| Left hippocampus | 0.43^a,b^ | 0.45^a^ | 0.43^a,b^ | 0.54 ^c^ | 0.51 | 0.46 |
| Right hippocampus | 0.45 ^a,b^ | 0.48 ^a,b^ | 0.47 ^a,b^ | 0.57 ^c^ | 0.55 | 0.50 |
| **Cerebral cortical folding** | | | | | | |
| Local gyrification index | 1.24 | 1.25 | 1.21 | 1.28 | 1.29 | 1.30 |
| Curvedness (mm^-1^) | 0.21^a^ | 0.21^a,b^ | 0.21 | 0.24 | 0.24 | 0.24 |
| Surface area (cm^2^) | 139.71 | 146.81 | 143.89 | 151.95 | 151.47 | 151.23 |

Results of least squares means from analysis of covariance, controlling for gestational age at scan, fetal sex, maternal weight at MRI.

^a^ Different from Unexposed fetuses with maternal EPDS ≤ 4 (adjusted p <0.05);

^b^ Different from Unexposed fetuses with maternal EPDS 5-9 (adjusted p <0.05);

^c^ Different from Unexposed fetuses with maternal EPDS ≥10 (adjusted p <0.05)

P values were adjusted for multiple testing based on the false discovery rate according to the Benjamini-Hochberg method.

Supplemental Table S4. Placenta measures in SRI-exposed group and unexposed controls with different prenatal maternal depression scales.

|  | **SRI-exposed** | | | **Unexposed** | | |
| --- | --- | --- | --- | --- | --- | --- |
| **EPDS** | **≤ 4** | **5-9** | **≥10** | **≤ 4** | **5-9** | **≥10** |
| Volume | 733.53 | 761.01 | 757.08 | 662.73 | 651.04 | 646.82 |
| ADC_D | 0.0037 | 0.0041 | 0.0031 | 0.0033 | 0.0034 | 0.0034 |

Results of least squares means from analysis of covariance, controlling for gestational age at scan and maternal weight at MRI.

Supplemental Table S5. Placenta measures in SRI-exposed group vs unexposed controls, excluding participants with prenatal alcohol exposure.

|  | **SRI-exposed** | **Unexposed** | **Difference (95% CI)** | **Adjusted P^a^** |
| --- | --- | --- | --- | --- |
| Volume | 754.63 | 653.55 | 101.08 (33.97 to 168.20) | 0.007 |
| ADC_D | 0.004 | 0.003 | 0.0004 (0.00002 to 0.0008) | 0.05 |

Results of least squares means from analysis of covariance, controlling for gestational age at MRI scan, maternal weight at MRI, and maternal depression score at MRI visit.

^a^ P values were adjusted for multiple testing based on the false discovery rate according to the Benjamini-Hochberg method.

Supplemental Table S6. Placenta measures in SRI-exposed group and unexposed controls with different prenatal maternal depression scales, excluding participants with prenatal alcohol exposure.

|  | **SRI-exposed** | | | **Unexposed** | | |
| --- | --- | --- | --- | --- | --- | --- |
| **EPDS** | **≤ 4** | **5-9** | **≥10** | **≤ 4** | **5-9** | **≥10** |
| Volume | 714.61 | 763.58 | 785.62 | 663.61 | 653.27 | 613.49 |
| ADC_D | 0.0037 | 0.0041 | 0.0032 | 0.0034 | 0.0034 | 0.0034 |

Results of least squares means from analysis of covariance, controlling for gestational age at scan and maternal weight at MRI.

Supplemental Table S7. Association between placenta measures and fetal brain volumes in SRI-exposed group.

| SRI-exposed | | Cortical gray matter | White matter | Deep gray matter | Cerebellum | Brainstem | Left hippocampus | Right hippocampus | Total brain volume |
| --- | --- | --- | --- | --- | --- | --- | --- | --- | --- |
| Volume | β | 0.0003 | 0.02 | 0.002 | 0.002 | 0.0005 | -6.73E-05 | 9.58E-05 | 0.05 |
|  | P value | 0.97 | 0.13 | 0.02 | 0.14 | 0.10 | 0.43 | 0.13 | 0.005* |
| ADC_D | β | 2493.92 | 6153.24 | 364.97 | 525.99 | -16.56 | -26.05 | 16.05 | 6886.81 |
|  | P value | 0.04 | 0.01* | 0.07 | 0.01* | 0.76 | 0.25 | 0.27 | 0.03 |

Results from General Linear Model, controlling for gestational age at MRI scan, fetal sex, maternal weight at MRI, and maternal depression score at MRI visit.

^*^Remained significant after adjusting for multiple testing.

Supplemental Table S8. Association between placenta measures and fetal cerebral cortical folding in SRI-exposed group.

| SRI-exposed | | Local gyrification index | Sulcal depth (mm) | Curvedness (mm^-1^) | Surface area (cm^2^) |
| --- | --- | --- | --- | --- | --- |
| Volume | β | 0.0001 | 0.0004 | -1.42E-05 | 0.03 |
|  | P value | 0.15 | 0.11 | 0.37 | 0.03 |
| ADC_D | β | 18.53 | 37.61 | -4.92 | 6595.05 |
|  | P value | 0.03 | 0.35 | 0.08 | 0.008 |

Results from General Linear Model, controlling for gestational age at MRI scan, fetal sex, maternal weight at MRI, and maternal depression score at MRI visit.
